# Harm reduction stories: leveraging graphic medicine to engage veterans in substance use services within the VA

**DOI:** 10.1186/s12954-023-00886-8

**Published:** 2023-12-06

**Authors:** Leah H. Harvey, Westyn Branch-Elliman, Jacqueline Boudreau, Samantha K. Sliwinski, Allen L. Gifford, Minh Q. Ho, Elizabeth Dinges, Justeen Hyde

**Affiliations:** 1 Department of Medicine, Section of Infectious Diseases, Boston University Chobanian & Avedisian School of Medicine and Boston Medical Center, 801 Massachusetts Avenue, 2nd Floor, Boston, MA 02118 USA; 2 Alpert School of Medicine at Brown University, Division of Infectious Diseases, Providence, RI USA; 3 Center for Healthcare Organization and Implementation Research, VA Boston Healthcare System, Boston, MA USA; 4 Harvard Medical School, Boston, MA USA; 5 Department of Medicine, Veterans Affairs Boston Healthcare System, Boston, MA USA; 6 Department of Medicine, Section of General Internal Medicine, Boston University Chobanian & Avedisian School of Medicine and Boston Medical Center, Boston, MA USA; 7 School of Public Health, Boston University, Boston, MA USA; 8 VA Orlando Health Care System, Orlando, FL USA; 9 VA Illiana Health Care System, Danville, IL USA

## Abstract

**Background:**

Harm reduction strategies can decrease morbidity and mortality associated with substance use. Various barriers limit conversation around substance use between clinicians and patients. Graphic medicine techniques can inform and encourage patient-centered conversations about substance use. We describe the co-development of a harm reduction-focused graphic medicine comic that depicts the infectious risks associated with injection drug use and patient-centered approaches to providing education about potential risk mitigation strategies.

**Methods:**

We formed a co-design group of veterans with lived experience with substance use, physicians, health services researchers, and community-based harm reduction leaders. Over the course of ten sessions, the co-design team developed a storyline and key messages, reviewed draft content and worked with a graphic designer to develop a comic incorporating the veterans’ input. During each session, co-design leads presented drafts of the comic and invited feedback from the group. The comic was edited and adapted via this iterative process.

**Results:**

The comic depicts a fictionalized clinical vignette in which a patient develops an injection-related abscess and presents to their primary care provider. The dialogue highlights key healthcare principles, including patient autonomy and agency, and highlights strategies for safer use, rather than emphasizing abstinence. Feedback from co-design group participants highlights lessons learned during the development process.

**Discussion:**

Graphic medicine is ideally suited for a patient-centered curriculum about harm reduction. This project is one of several interventions that will be integrated into VA facilities nationally to support incorporation of harm reduction principles into the care of persons who inject drugs.

**Supplementary Information:**

The online version contains supplementary material available at 10.1186/s12954-023-00886-8.

## Introduction

Harm reduction strategies, including safe injection education and efforts to engage people who use drugs (PWUD) in care, are vital to decreasing the morbidity and mortality associated with substance use. Harm reduction typically refers to practical, person-centered strategies to reduce potential harms associated with drug use, such as overdose or infection [1]. Examples include naloxone distribution for overdose prevention, syringe service programs for infection prevention, and low-barrier access to medications for opioid use disorder (MOUD) to facilitate patient engagement in care and reduce mortality [2–4]. Although harm reduction strategies are evidence-based, uptake has been limited in many clinical settings.

Exploratory interviews conducted with providers who care for PWUD within a sample of Veterans Affairs Medical Centers found variable understandings of and beliefs about harm reduction and barriers and facilitators to integrating and expanding harm reduction into the continuum of care [5, 6]. Three key barriers were identified: limited provider knowledge and experience with harm reduction and substance use-focused care, limited patient engagement, and stigma around substance use. To address these identified implementation barriers, we sought to develop and pilot an innovative tool intended to engage and educate both patients and providers.

Graphic medicine refers to the use of comics and visual storytelling to personalize and illustrate a medical topic, often by chronicling an individual’s experience [7]. Given its intrinsic ability to visually explain and humanize complex topics, graphic medicine comics have been used successfully in public health campaigns and in medical education [8–10]. Patients and their families have also produced poignant graphic medicine illness narratives depicting their experience undergoing chemotherapy or pregnancy, for example, that highlight the emotional impact of clinical care, including misconceptions about disease and treatment and frustrations navigating the healthcare system [11, 12]. Prior published work also underlines the utility of combining pictures and text to enhance understanding via different neural pathways, which can also improve memory and recall, especially among those with limited literacy [9, 13]. Recently, the COVID pandemic inspired many graphic medicine comics, including both educational comics depicting viral transmission and personal, more therapeutic comics describing individuals’ experience with isolation and loss [14–16].

Stemming from the early days of the harm reduction movement, graphic medicine has also been utilized by PWUD to educate and advocate for themselves and each other. Graphic medicine comics were often included as part of ‘zines’ or other informal publications and circulated within communities [17]. More recently, harm reduction organizations in many regions have supported the development and distribution of graphic medicine comics focused on aspects of substance use, including overdose prevention, wound care, risks associated with polysubstance use, and overdose-associated grief [18–21]. To our knowledge, however, there is limited published literature describing the development of graphic medicine comics, discussing doctor-patient conversations about substance use, or focusing on substance use with the veteran community. To that end, we describe the collaborative development of a harm reduction-focused graphic medicine comic working with a co-design team composed of veterans, clinicians, community-based harm reduction leaders, and health services researchers.

## Methods

Our graphic medicine comic utilizes a fictionalized clinical vignette to depict the infectious risks associated with injection drug use, identify potential risk mitigation strategies, and model patient-centered discussions around substance use in the outpatient clinical setting. The co-design project was submitted to the lead institution’s institutional review board for review and was determined to be exempt. Following this determination, review was waived at secondary sites.

### Co-design team

A major aim of the creation of the graphic medicine comic was to maximize cultural competency and collaboration. To that end, we recruited a co-design group comprised of veterans with lived experience with substance use (either current or past), four clinicians, two community-based harm reduction leaders, and three health services researchers. Veteran group members were recruited via flyers and personal outreach conducted by clinicians and researchers. Ten veterans initially expressed interest in participating but two dropped out of the group in the early stages of the project due to health issues. The remaining eight veteran members represented a variety of ages, races, ethnicities, geographic regions, and substance use statuses. The illustrations in the graphic medicine comic were developed by a graphic designer who designed and piloted his work in collaboration with the co-design team.

### Approach

**Fig. 1:**
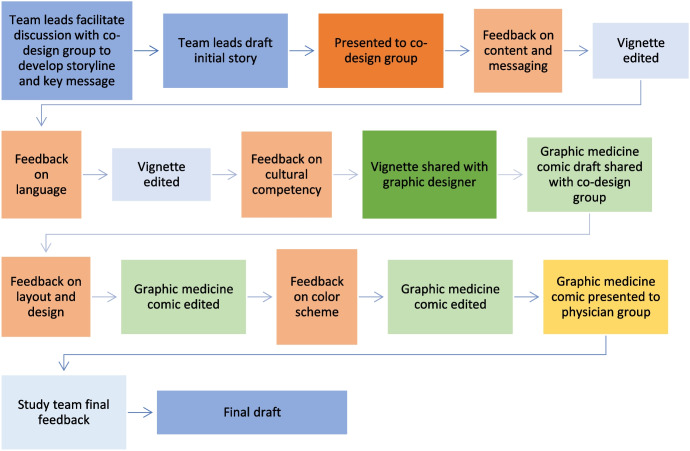
Graphic Medicine Comic Co-Design Process

Over the course of ten one-hour-long sessions over a six month period, the co-design team reviewed draft content, including the vignette and educational material discussing infection prevention techniques and existing VA resources, and worked with the graphic designer to develop a comic incorporating the veterans’ input. During initial sessions, the group discussed storylines and key messages to be communicated. Between sessions, the lead facilitation team, comprised of a clinician and a researcher, drafted content based on group’s input. Content was shared ahead of meetings and feedback from the group was discussed during meetings. At least two members of the facilitation team took detailed notes during each session to document participant feedback and comments. The comic was edited and adapted via this iterative process. Each session focused on a topics relevant to the stage of product development, such as key harm reduction messaging and storyline during initial meetings, representation and character development and language used to describe substance use in mid-course meetings, and color schemes and design decisions in later meetings. See Fig. 1 for overview of process.

Near final drafts of the comic were also shared with a group of physicians, including infectious disease specialists and hospitalists. Feedback was shared with the co-design team and used to inform final edits to language and images.

## Results

The graphic medicine comic, titled ‘*Your Health Matters: Let’s Talk About How To Stay Safe While Using,*’ is built around a fictionalized vignette of a veteran with opioid use disorder who develops an injection-related abscess and seeks care at his primary care physician (PCP)’s office. We chose this scenario to portray a common, realistic, and straightforward clinical situation that would be applicable to a range of patients, provided an appropriate and opportune moment for teaching, and could model dialogue of a patient-centered conversation about substance use.

In the vignette, the veteran is initially hesitant to disclose that his abscess is related to injection drug use. The PCP uses non-judgmental, person-centered communication skills to build rapport and a sense of safety for the patient to discuss his substance use. The PCP’s use of factual, non-stigmatizing language then facilitates further discussion about harm reduction principles and techniques for safer injection. The dialogue is intended to emphasize key messages or themes, including patient autonomy and patient agency, model open communication techniques, and highlight strategies for safer use, rather than emphasizing abstinence. (Additional file 1).

**Fig. 2:**
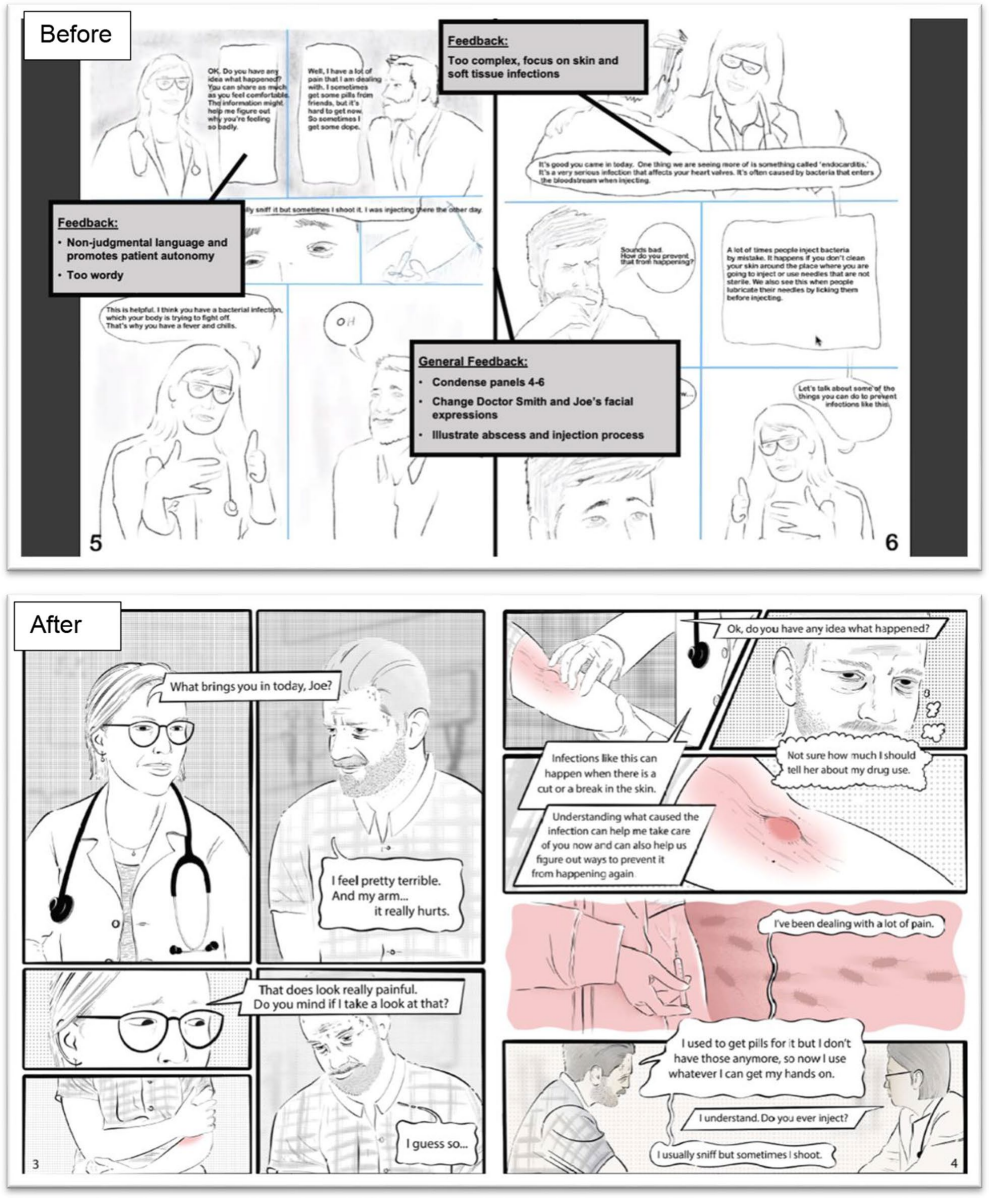
Drafts of Graphic Medicine Comic Before and After Incorporating Co-Design Group Feedback

The graphic medicine comic was written and revised via an iterative design process to explore the extent to which it achieved the intended goals of improving knowledge about infectious risks and risk mitigation strategies associated with injection drug use and resources within the VA available to PWUD. Figure 2 contains drafts of sample panels in the graphic medicine comic and demonstrates the revision process and incorporation of group feedback.

Feedback from the veteran members of the co-design group and the physician group was critical for developing educational messaging. In the initial stages of this project, the study team drafted the outline of the vignette and key messaging that we thought would be important to emphasize in the comic. This messaging was adapted and clarified based on input from the co-design and clinical groups. The clinical group was asked to reflect upon the comic’s tone, clinical accuracy, and overall effectiveness, including whether the physicians would consider utilizing the book in their own practice, and the comic was modified to reflect their input.

Table 1 describes the lessons learned and how co-design group input was incorporated during the design process. One recurring recommendation from the co-design group was the importance of simplified storytelling. The first iteration involved a more complex story of a patient with injection-associated infective endocarditis and an epidural abscess. This was eventually simplified to be a soft tissue infection, the underlying mechanism of which was more straightforward to convey. Messaging about safer injection techniques, overdose prevention, infection screening and prevention, and other topics was also streamlined to facilitate communication of key points, avoid dense and distracting detail, and maintain interest.

**Table 1:** Co-design group feedback and lessons learned

Key messaging	Selected feedback from co-design group*	Depiction in graphic medicine comic
Patient autonomy regarding disclosure of substance use	*I don’t know how many people would open up to their doctor. I opened up to mine. Doctor needs to be responsible and non-judgmental. Maybe they are thinking about opening up and if they see that their doctor is reacting like the doctor in the story they might. (Veteran)*	Promoting patient agency and autonomy in their own care
		Emphasis on rapport and trust-building between patient and provider
Patient-centered dialogue and nonverbal communication skills can build trust	*It’s also important how the doctor presents himself. Eye contact; are you looking at the screen? Are you warm? Are you cold? Are you being a doctor or are you being a person who is someone you can open up to? (Veteran)*	Avoiding accusatory or judgmental language around substance use
		Illustrating provider eye contact, body language, and active listening skills
Physicians are willing and able to engage in patient-centered conversations, even if they have limited experience with substance use	*Providers wanting to ask questions but they didn’t know where to start; we could not get anywhere. What made the difference, if we can just get this first thing, then that gave me the opportunity to engage. (Veteran)*	Use of open-ended questions to encourage dialogue
	*Yeah, I’m open to it; I just didn’t know how to start or what to say. (Physician)*	Provider listens to patient before offering thoughts
Patients and providers can learn from each other about substance use and associated risks	*The line ‘let’s talk more about that’ always tends to reel me in. Listen to me a little more. The more they listen to me, the more I am giving out information every word I say. (Veteran)*	Provider asks permission before providing education
	*Medical doctors always made me feel in fear that they knew me better than I did. That book smart stuff scares me off. Doctors have to listen and hear, listen and hear- that’s what develops trust. (Veteran)*	Provider uses simple language to explain concepts and offers opportunities to ask questions
Use of non-stigmatizing language to describe substance use and PWUD	*I see I am going to have to change my terms. Stigma plays a large role in the recovery process. I wasn’t even aware I was using stigmatizing terms; it just became ingrained. (Veteran)*	Both patient and provider model use of non-judgmental, non-stigmatizing language
Defining key clinical terms, such as endocarditis	*Yeah, I never heard much about bacteria [sic] infections when I was using. And endocarditis? Forget it. (Veteran)*	Clinical complexity in vignette simplified to injection-associated abscess
	*We just assume that we’re speaking the same language and how A can lead to B, but we’re not. (Physician)*	Discussion of how inadvertent injection of bacteria can cause both skin infections and more invasive infections
Public health messaging can be dynamic and engaging	*For this, maybe don’t throw too many messages. Maybe could throw something very brief in but could turn into too much of a stew of different things. (Veteran)*	Streamlining vignette to focus on safer injection techniques
Harm reduction involves multiple complementary strategies, including MOUD, infection screening, safe injection techniques, naloxone, and not using alone	*Felt a little long and complicated for your average person, a lot of words. Too much in one booklet… The message is clear, but you might lose people before you get to the important part—here’s how you get help. (Physician)*	Other harm reduction strategies featured in centerfold of graphic medicine comic rather than incorporated into vignette
Emphasis on harm reduction, not necessarily abstinence from substance use	*Yeah, we remind people that any substance could be tainted with fentanyl… it’s a different game nowadays. That made me want to quit but maybe not everybody. (Veteran)*	Provider offers patient MOUD and emphasizes that it is entirely voluntary and his care will not be impacted if he declines
Substance use can affect everyone and thus characters depicted in the graphic medicine comic should represent a diversity of identities	*We’re scrolling, and nothing looks like me. Maybe we could make the buddy a little more diversified. (Veteran)*	More defined demographic characteristics of graphic medicine comic characters
		Representation of multiple races, ethnicities, ages, genders, and branches of the military
Graphic medicine comic format is engaging	*I like the comic style but does that make light of the message? Like does it need to be more serious? (Veteran)*	Realistic illustrations and changes to graphics and color scheme to de-emphasize comic style
Peer-to-peer education can be an important component of harm reduction and recovery	*If you educate the right person, it gets back to the streets and they share the right information…When there is important information they spread it. (Veteran)*	Graphic medicine comic ends with a ‘teaching it forward’ moment between two friends
	*When I do hear support, I tell people my story. It breaks the ice to get rid of barriers. Then I tell them where I am now. (Veteran)*	

*Comments were documented near verbatim through detailed notetaking during meetings

*MOUD* Medications for opioid use disorder

## Discussion

Strategies to address stigma around substance use, improve patient engagement, and expand provider capacity around substance use-focused care remain important challenges in healthcare. Although education is a key component of addressing stigma and provider capacity to provide appropriate care, prior work has demonstrated that targeted educational interventions do not always translate into sustained clinical behavior change and that more integrated strategies may be required to achieve desired change. [22–24] This graphic medicine comic was co-developed by a group of veterans with lived experience, clinicians, harm reduction leaders, and health services researchers to facilitate informed and patient-centered conversations around substance use and harm reduction. It is our hope that this comic will appeal to and educate both patients and providers and that it be utilized as part of a comprehensive bundle of harm reduction programming.

As prior work demonstrates, graphic medicine can be a useful tool to elucidate and humanize complex issues and to inspire behavior change. In contrast to other qualitative tools, one advantage of graphic medicine is that it can be fictionalized and de-personalized, thus avoiding issues around privacy that can arise with potentially stigmatizing issues. [9] The narrative format models open communication techniques around a sensitive topic, which can engage and educate both patients and providers.

There are limitations to this pilot project. The graphic medicine comic was developed with a co-design group of veterans whose perspectives may not necessarily reflect that of all veterans or non-veteran populations. Likewise, the graphic medicine comic has been reviewed by a limited number of clinicians and harm reduction specialists and would benefit from further evaluation in other settings and experts in other fields. In the future, we intend to test this graphic medicine comic as an implementation science strategy to determine if the comic improves patient and provider comfort around discussing substance use and whether improved comfort translates into improved clinical outcomes.

In conclusion, graphic medicine is an engaging, accessible, and underused health communication method well-suited for a patient-centered curriculum about harm reduction strategies. Results from this project will inform the development of a more comprehensive bundle of harm reduction interventions that will be integrated into VA facilities nationally. In line with open-source science principles, the graphic medicine materials are included as a supplementary file available for download and use.

### Supplementary Information

**Additional file 1**: Graphic medicine comic.

## Data Availability

Graphic medicine comic is available as a supplement
