# Supplementary material for: Harm reduction stories: leveraging graphic medicine to engage veterans in substance use services within the VA
Source: Harm Reduct J. 2023 Dec 6;20:177. doi: 10.1186/s12954-023-00886-8 (PMC10702088; doi:10.1186/s12954-023-00886-8)
Supplement: Supplementary file 1 — Additional file 1: Graphic medicine comic. [file 12954_2023_886_MOESM1_ESM.pdf]

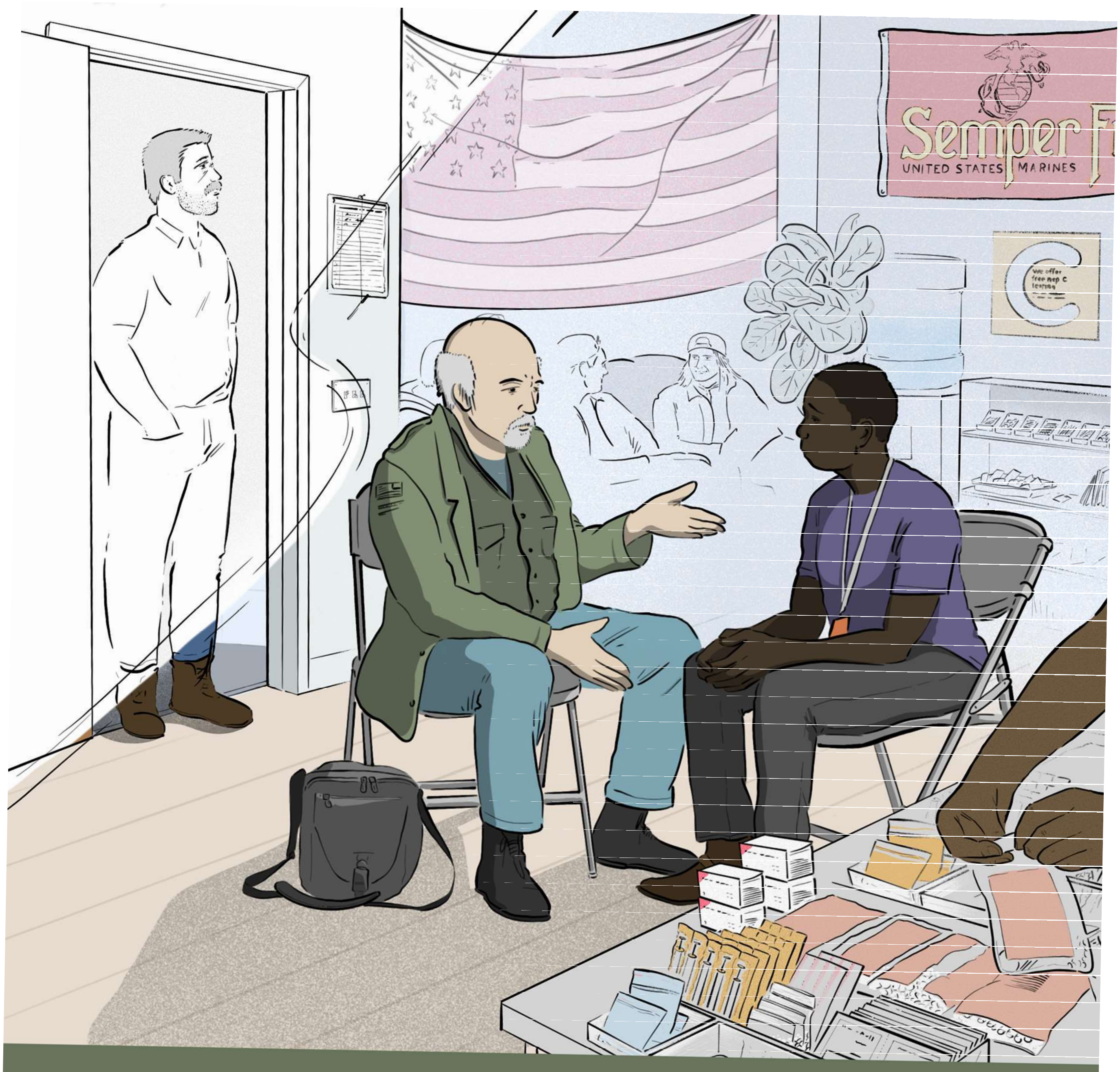

YOUR HEALTH MATTERS

Let's Talk About How to Stay Safe While Using

VA | U.S. Department of Veterans Affairs

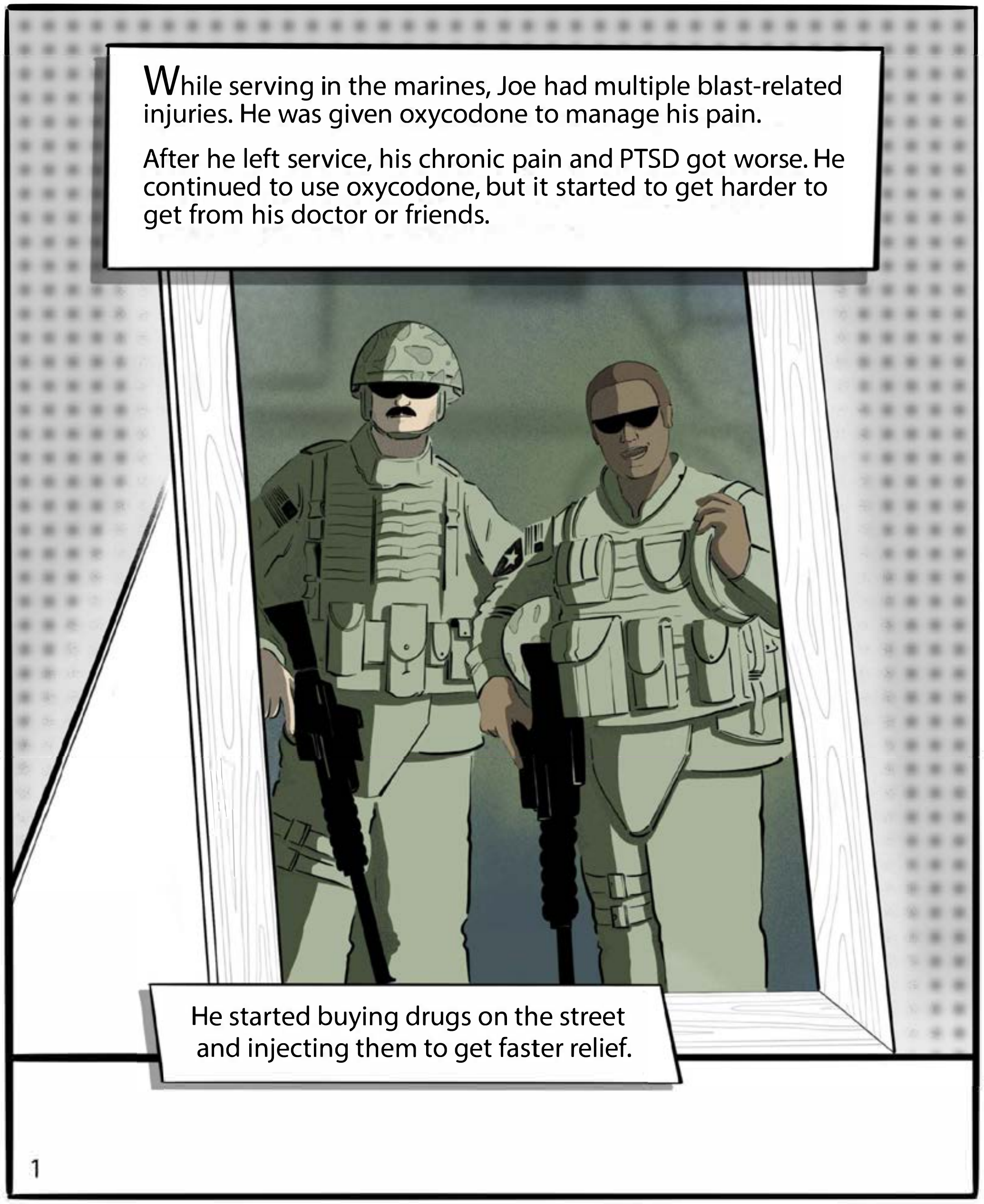
While serving in the marines, Joe had multiple blast-related injuries. He was given oxycodone to manage his pain.
After he left service, his chronic pain and PTSD got worse. He continued to use oxycodone, but it started to get harder to get from his doctor or friends.
He started buying drugs on the street and injecting them to get faster relief.

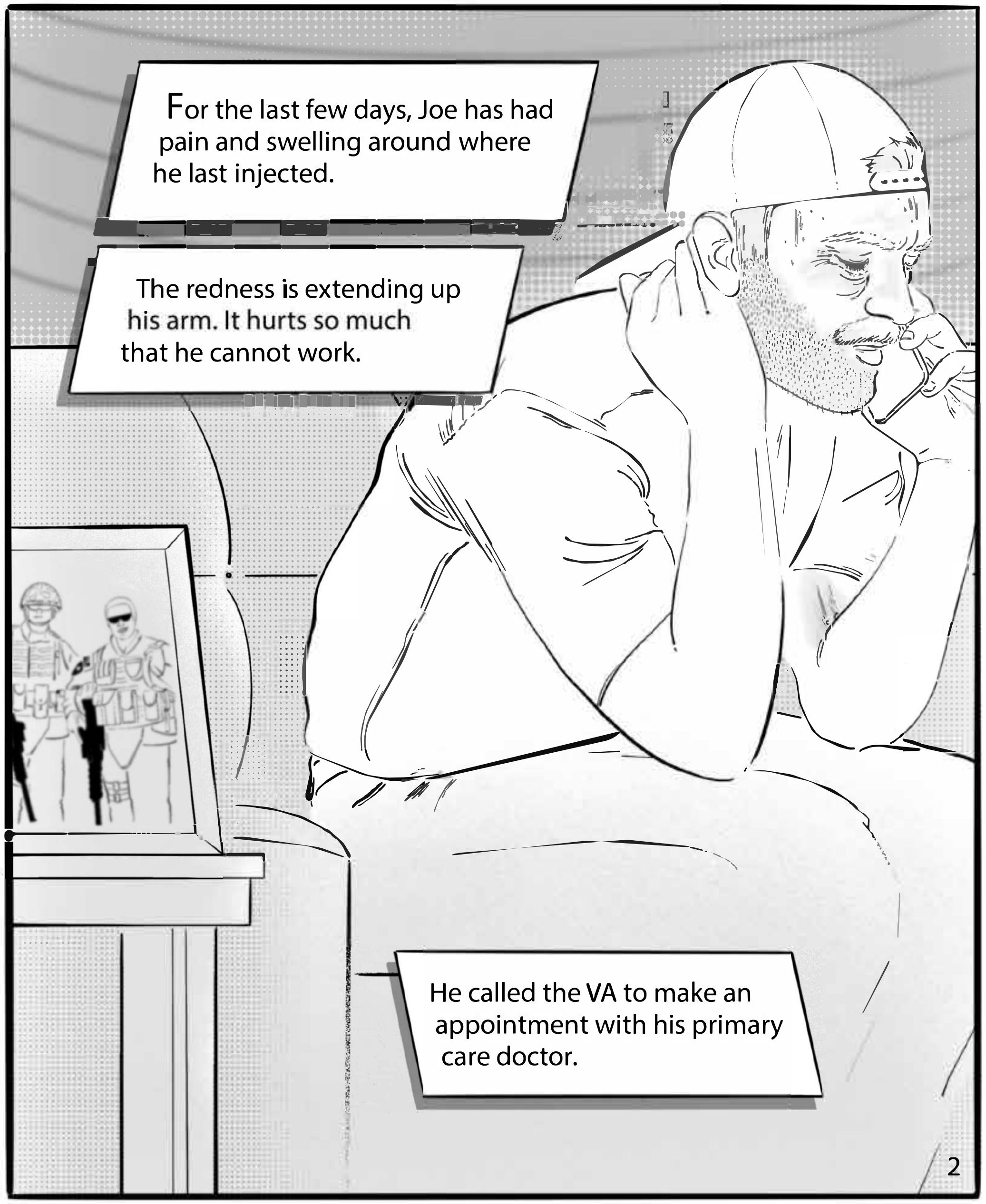
For the last few days, Joe has had pain and swelling around where he last injected.
The redness is extending up his arm. It hurts so much that he cannot work.
He called the VA to make an appointment with his primary care doctor.

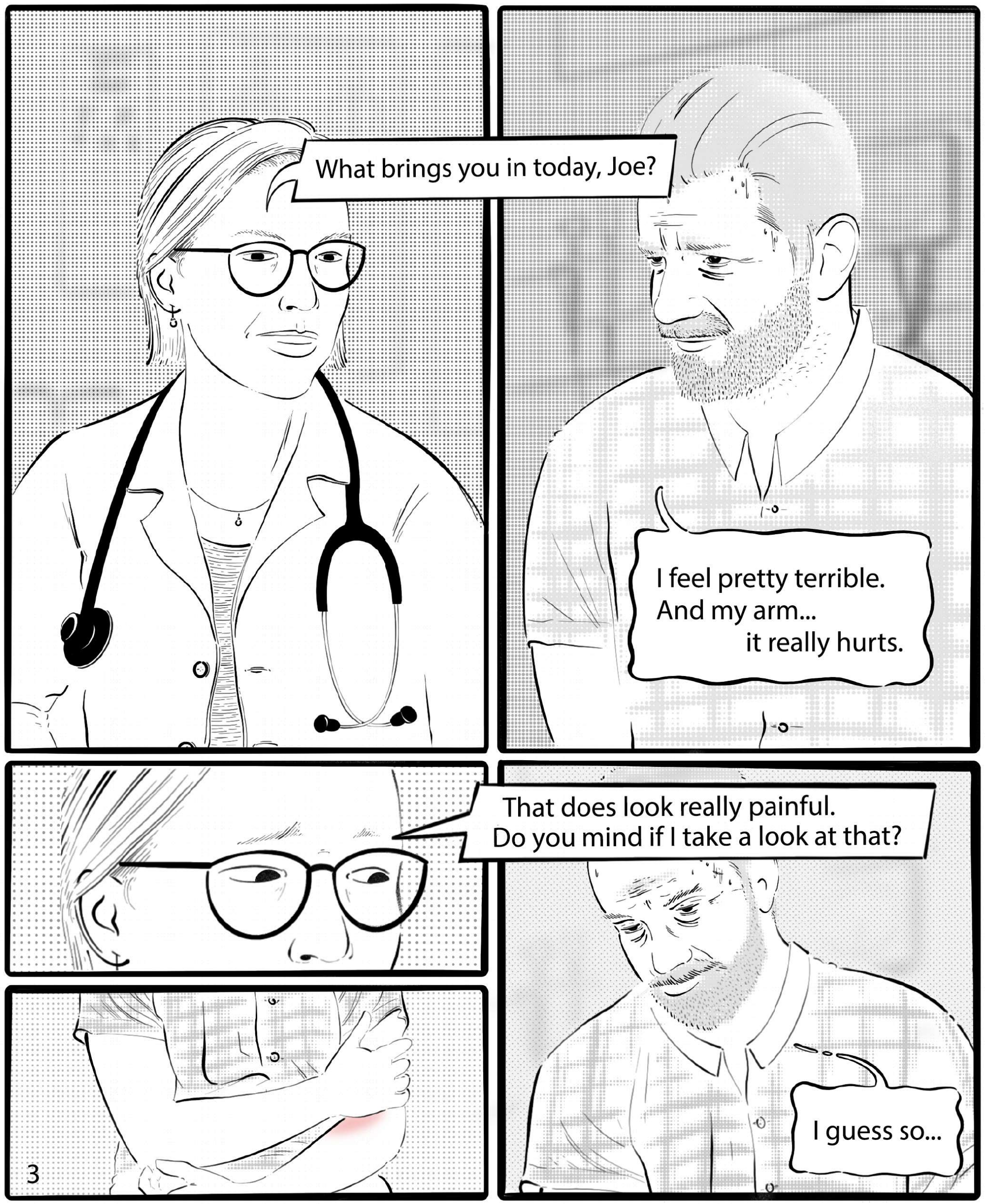
What brings you in today, Joe?
I feel pretty terrible. And my arm... it really hurts.
That does look really painful. Do you mind if I take a look at that?
I guess so...

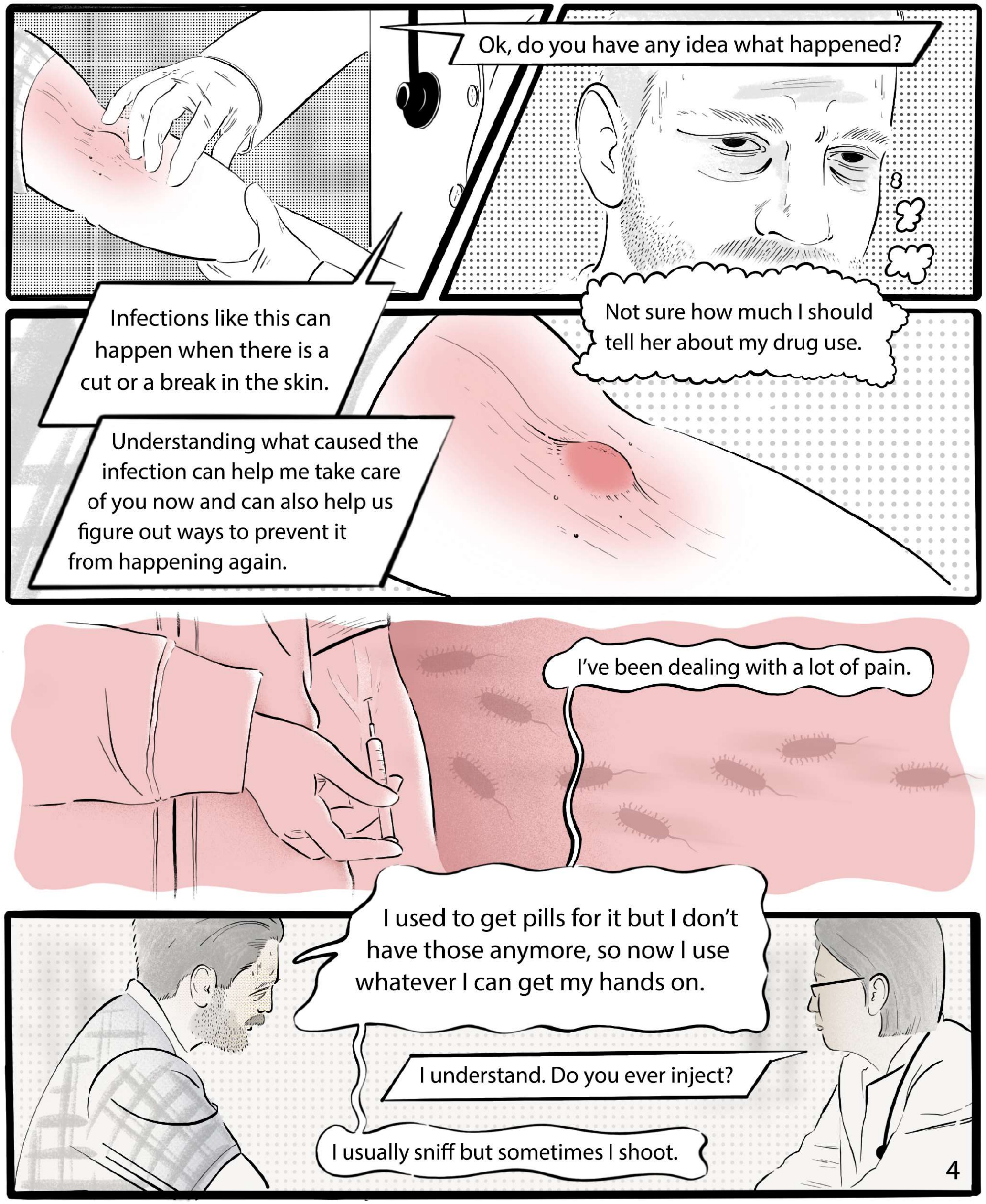
Ok, do you have any idea what happened?
Infections like this can happen when there is a cut or a break in the skin.
Not sure how much I should tell her about my drug use.
Understanding what caused the infection can help me take care of you now and can also help us figure out ways to prevent it from happening again.
I've been dealing with a lot of pain.
I used to get pills for it but I don't have those anymore, so now I use whatever I can get my hands on.
I understand. Do you ever inject?
I usually sniff but sometimes I shoot.

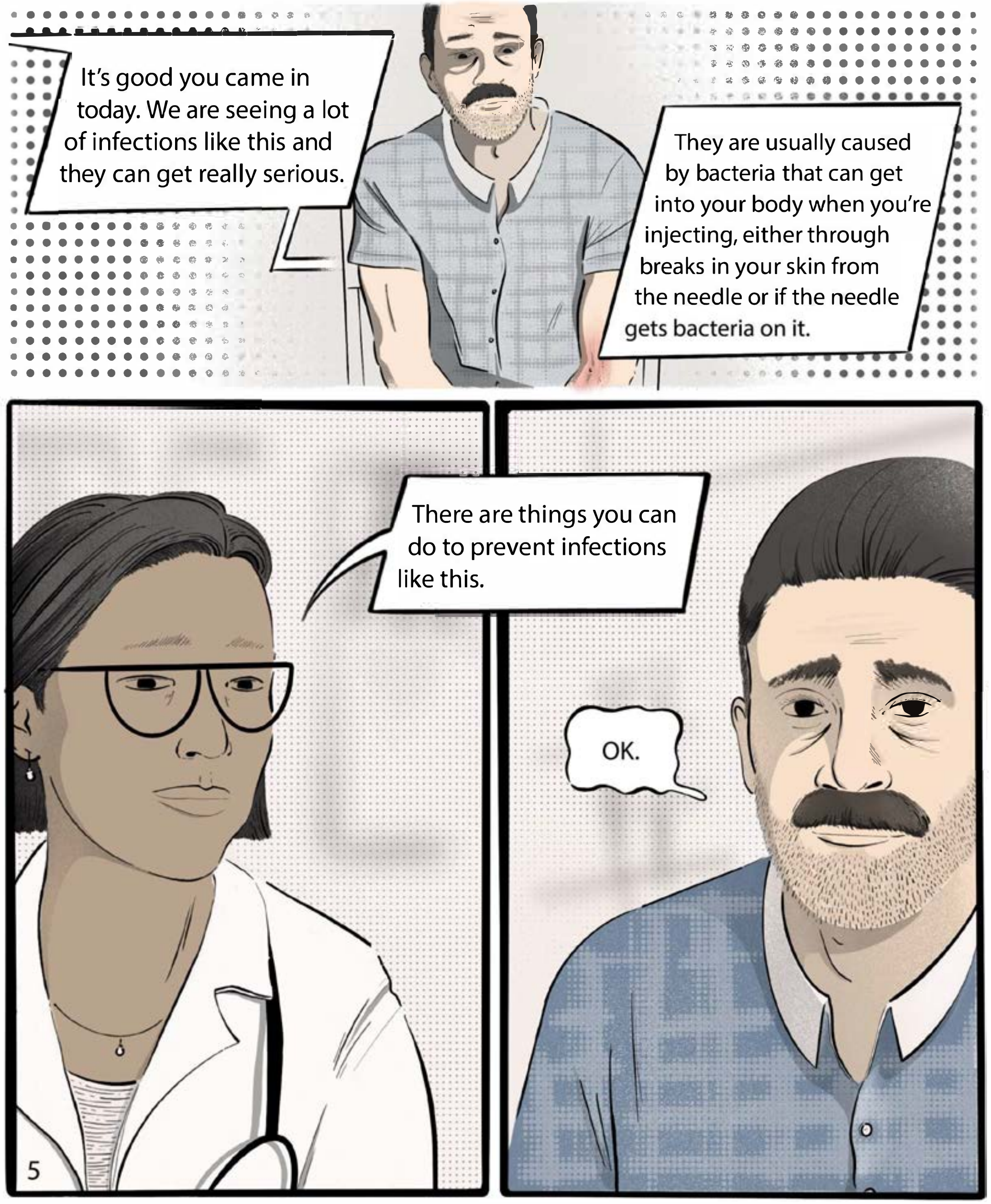
It's good you came in today. We are seeing a lot of infections like this and they can get really serious.
They are usually caused by bacteria that can get into your body when you're injecting, either through breaks in your skin from the needle or if the needle gets bacteria on it.
There are things you can do to prevent infections like this.
OK.

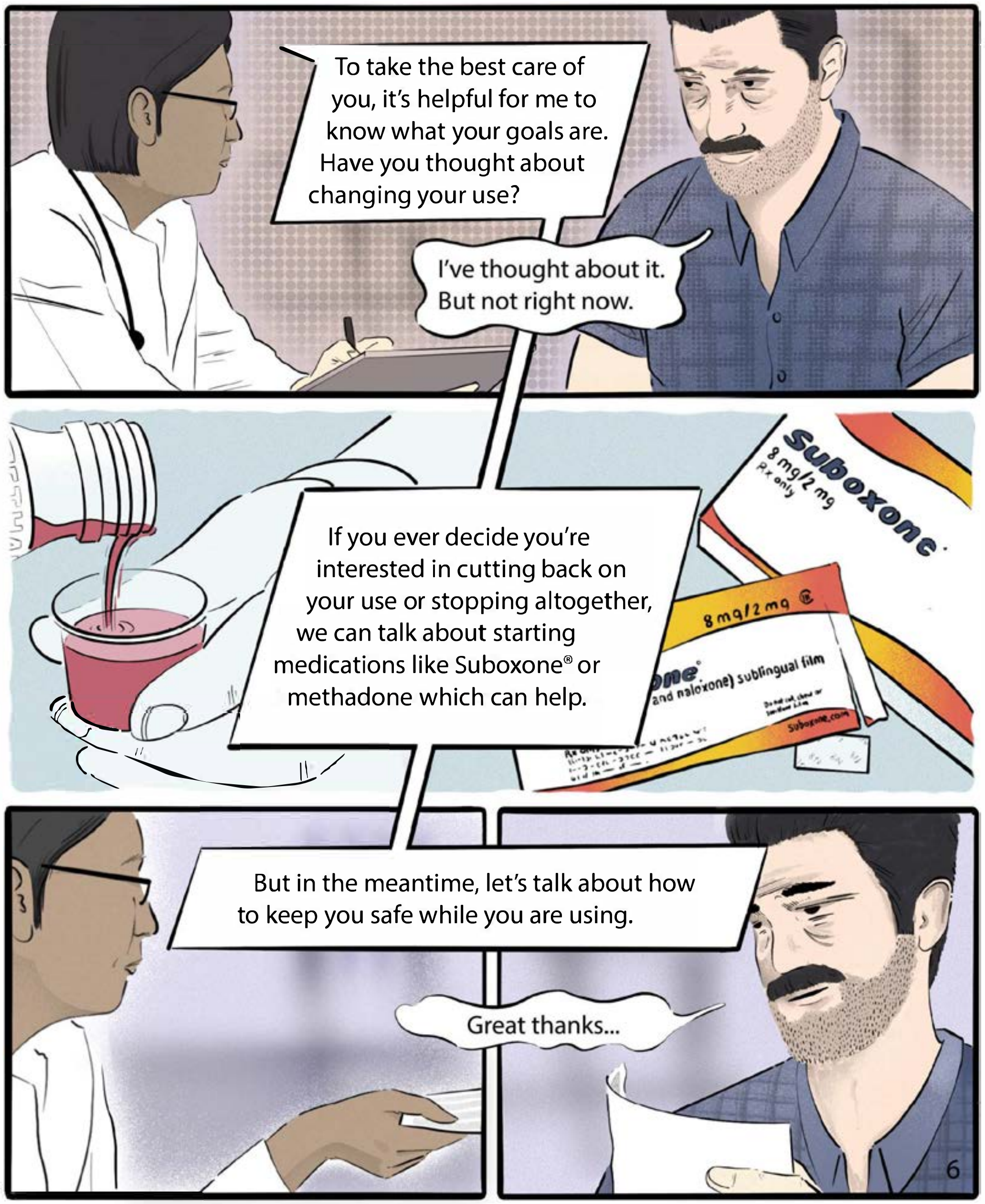
To take the best care of you, it's helpful for me to know what your goals are. Have you thought about changing your use?
I've thought about it. But not right now.
Suboxone
8 mg/2 mg
Rx only
8mg/2mg
and naloxone) sublingual film
If you ever decide you're interested in cutting back on your use or stopping altogether, we can talk about starting medications like Suboxone® or methadone which can help.
But in the meantime, let's talk about how to keep you safe while you are using.
Great thanks...

To Reduce the Risk of an Overdose

Always have Naloxone on hand when you use in case you or someone else overdoses

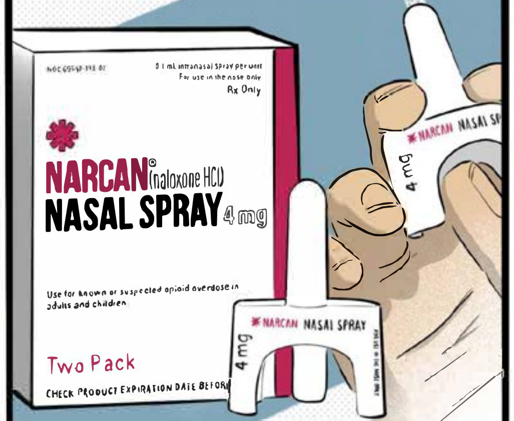

If you can, test your drugs before you use them

- Start with a small amount first to test what you're using
- Fentanyl test strips can also help you learn what is in your drugs
- Even drugs like methamphetamine, cocaine, and pressed pills pose a risk for overdose because they can contain fentanyl and other drugs

Don't inject alone

- Use with others
- Call the ***Never Use Alone*** line @ 800-484-3731 or ***www.neverusealone.com***

To Prevent Viral and Bacterial Infections

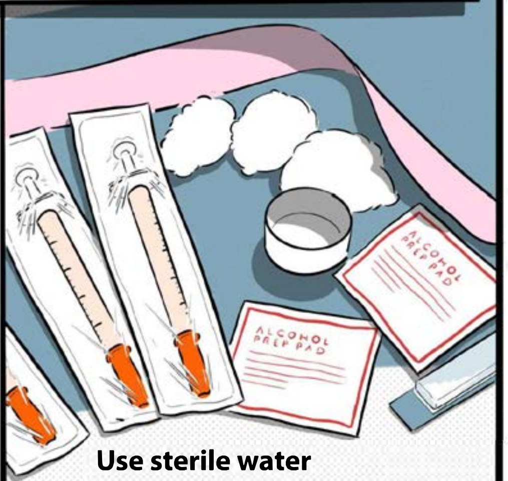

Use sterile water

- Use sterile water. Water can also be sterilized by boiling it in a clean cooker

Use new, sterile needles and supplies EVERY TIME you inject

- Used needles, syringes, cookers, and cottons can be coated with bacteria and viruses.
- You can get infections when bacteria and viruses get into the blood stream

Clean the skin around where you are planning to inject

- Alcohol swab is best
- Soap and water or hand sanitizer are okay, too

Keep your needle and syringe clean right up until you inject

To help Prevent the Spread of Infectious Diseases

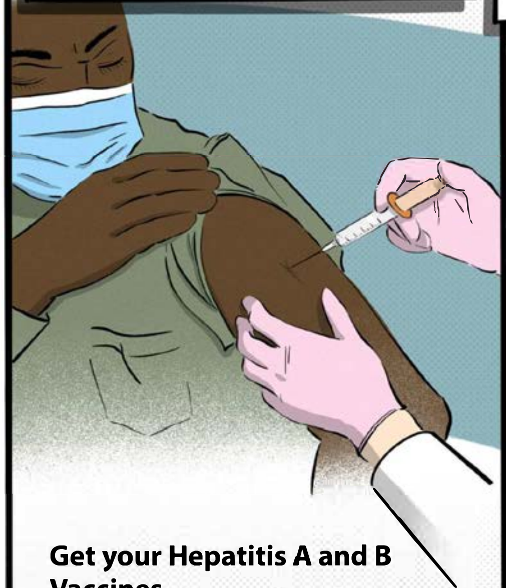

Get your Hepatitis A and B Vaccines

- These will protect you from these infections in the long run

Test regularly for HIV and Hep C

- Early identification and treatment will help you and others by preventing the spread of these infections
- You can get Hep C more than once. Even if you are tested and treated, it is important to keep getting tested

Dispose of your needles in a sharps container or other sturdy container

To Protect Your Veins and Prevent Other Health Problems

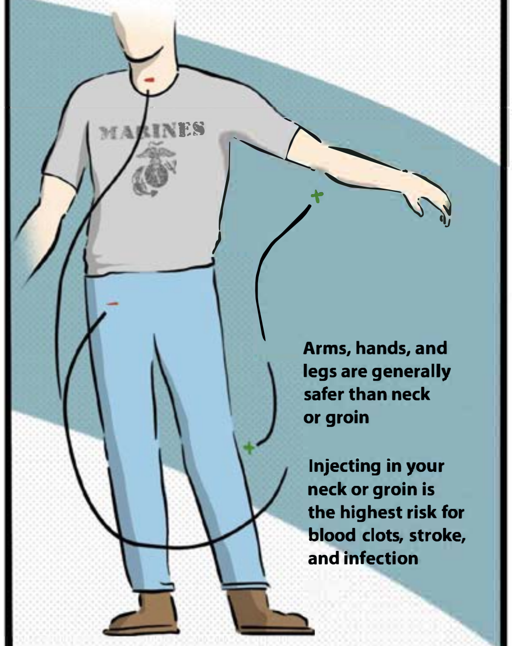

Use a new needle every time

- Used needles get dull quickly and can cause more damage to your skin

Try to inject in parts of your body that are lower risk for causing harms, like infection and stroke

- And consider changing how you use every once in a while to give your veins a break

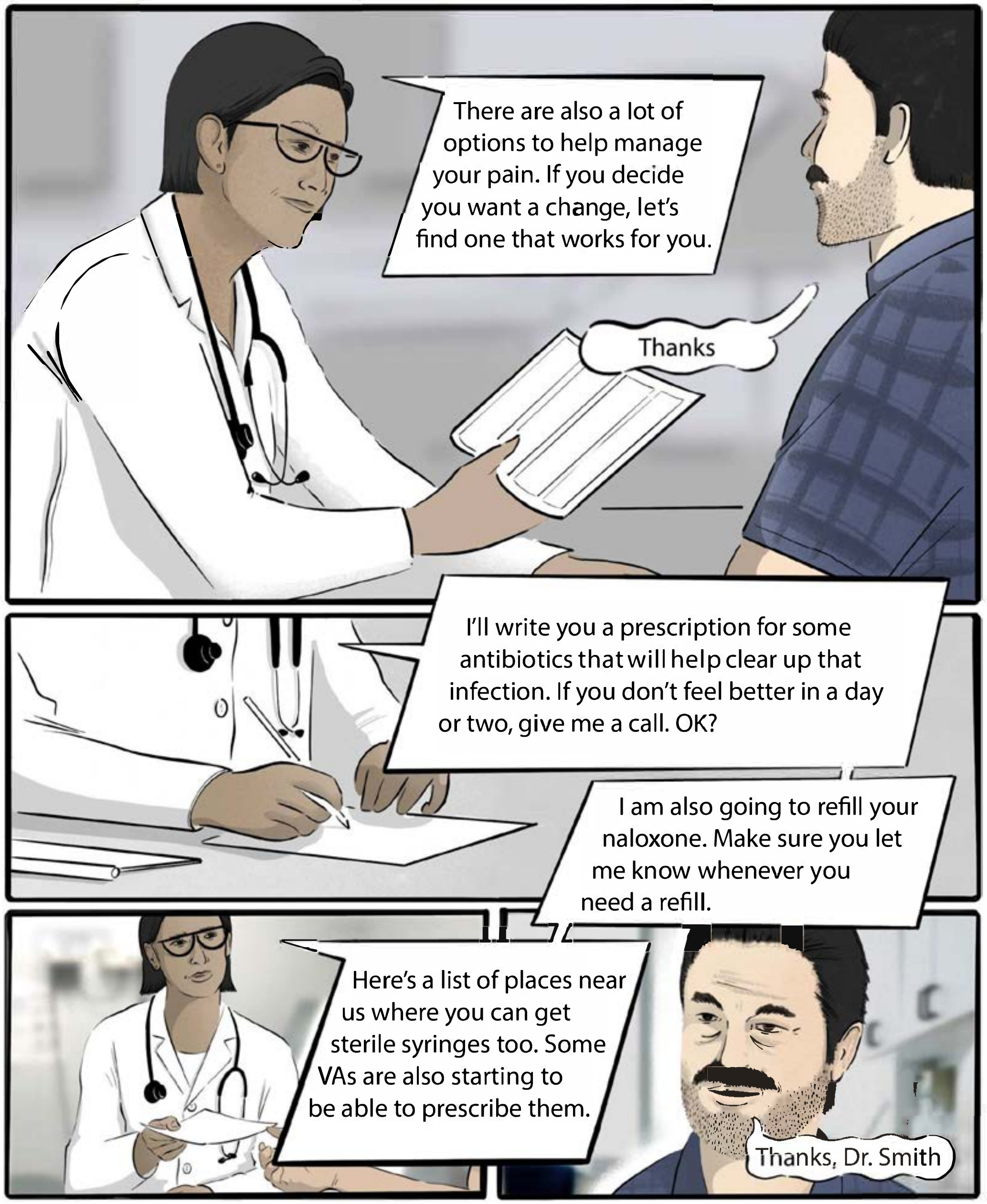
There are also a lot of options to help manage your pain. If you decide you want a change, let's find one that works for you.
Thanks
I'll write you a prescription for some antibiotics that will help clear up that infection. If you don't feel better in a day or two, give me a call. OK?
I am also going to refill your naloxone. Make sure you let me know whenever you need a refill.
Here's a list of places near us where you can get sterile syringes too. Some VAs are also starting to be able to prescribe them.
Thanks, Dr. Smith

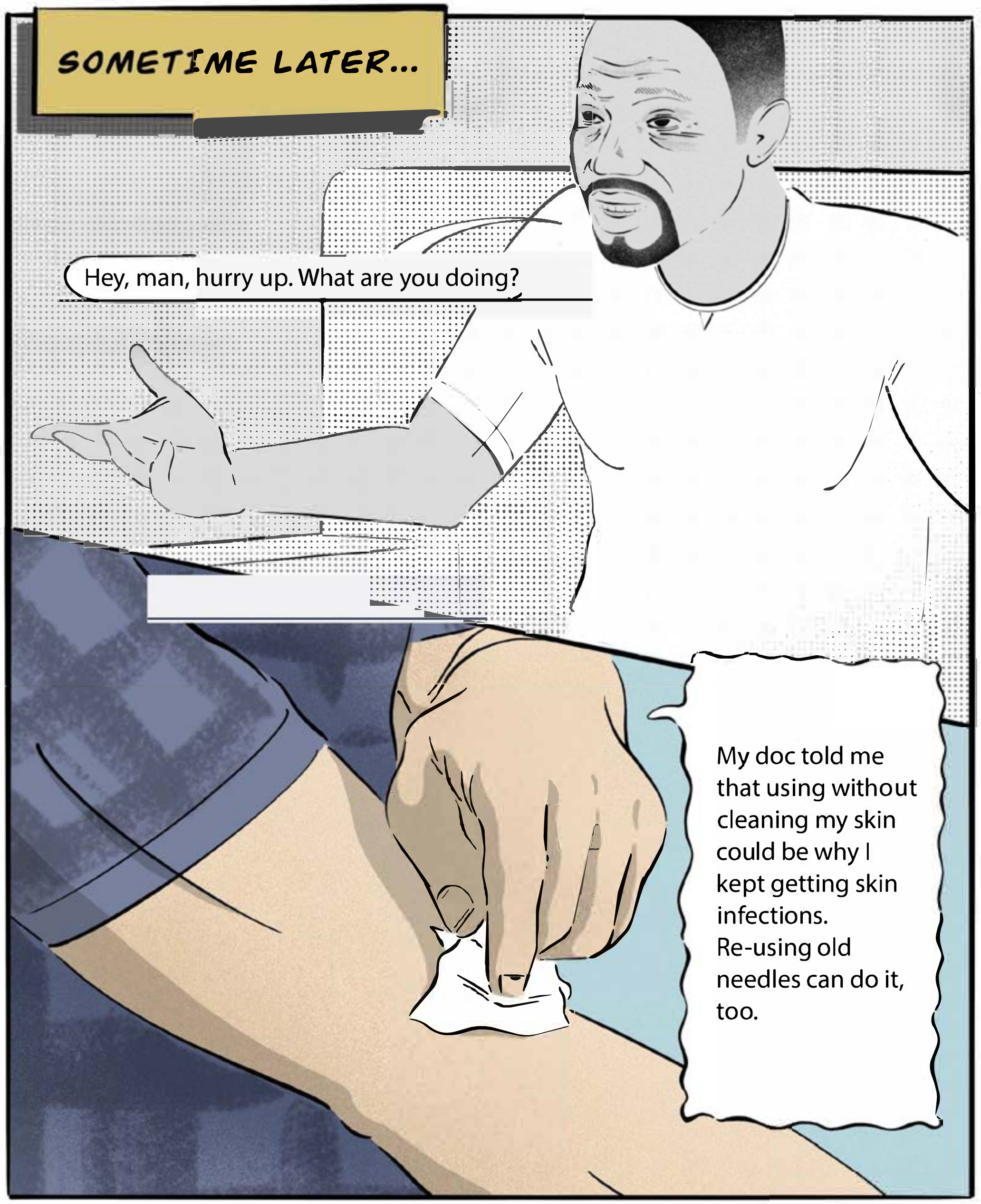
SOMETIME LATER...
Hey, man, hurry up. What are you doing?
My doc told me that using without cleaning my skin could be why I kept getting skin infections. Re-using old needles can do it, too.

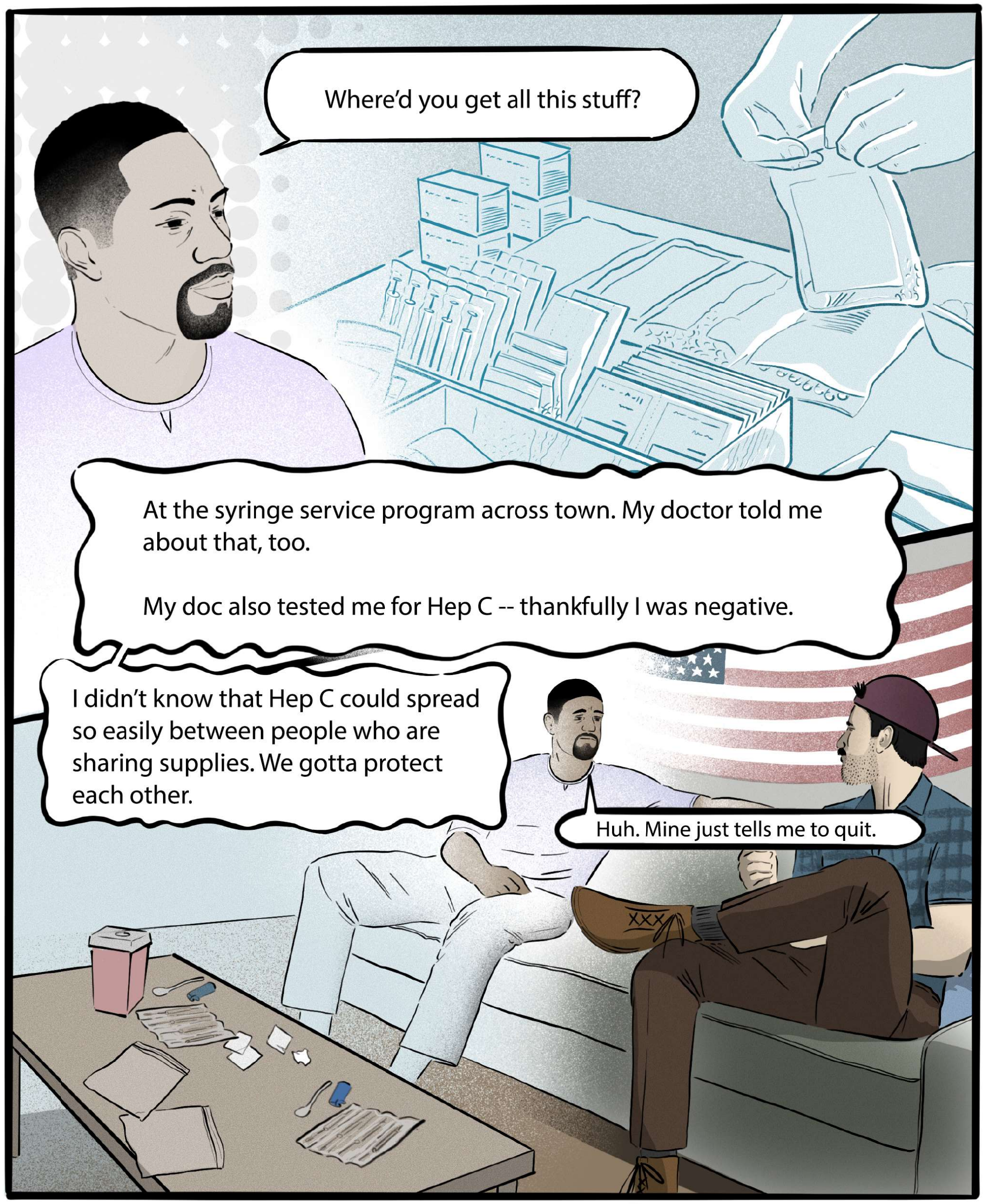
Where'd you get all this stuff?
At the syringe service program across town. My doctor told me about that, too.
My doc also tested me for Hep C -- thankfully I was negative.
I didn't know that Hep C could spread so easily between people who are sharing supplies. We gotta protect each other.
Huh. Mine just tells me to quit.

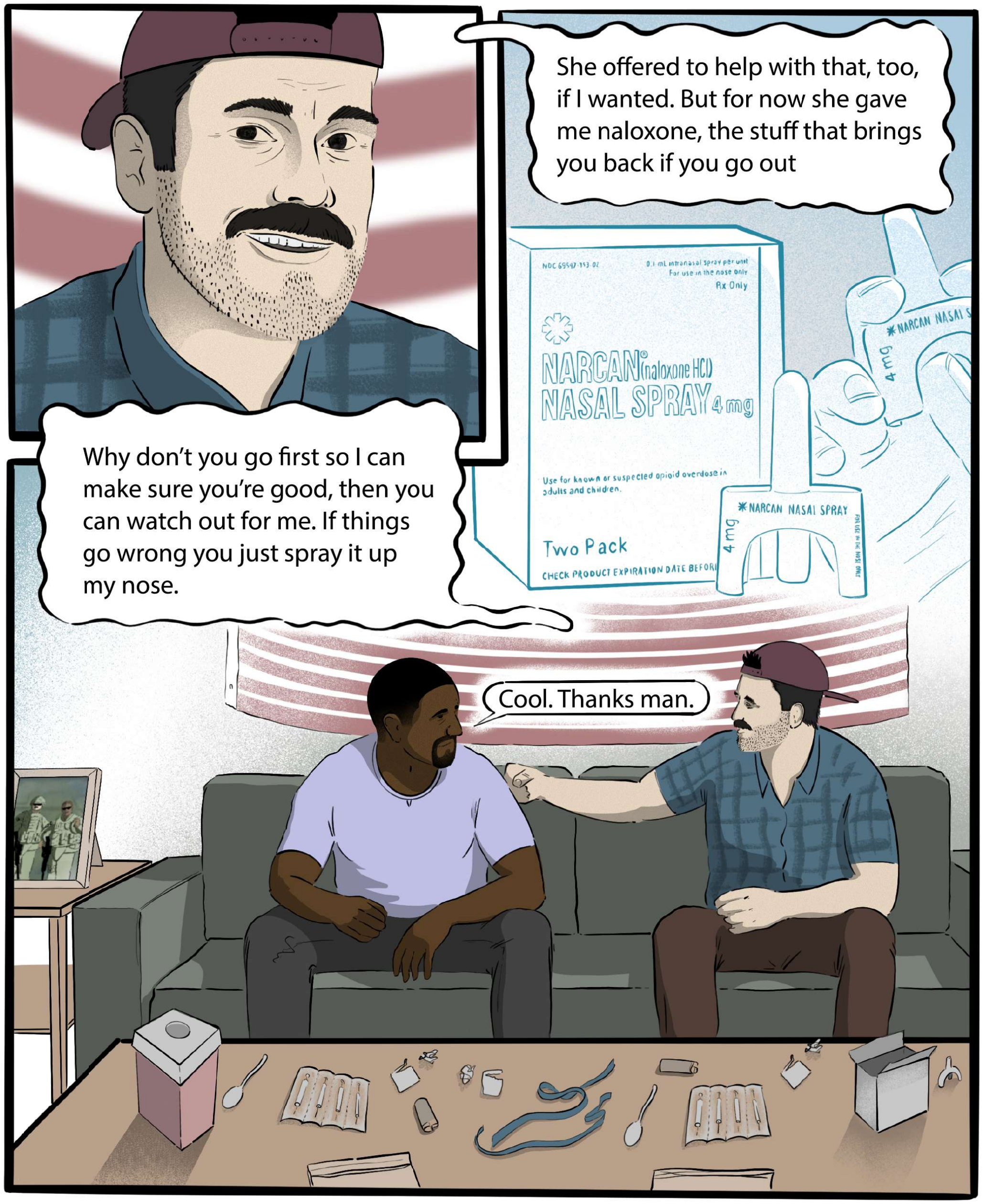
She offered to help with that, too, if I wanted. But for now she gave me naloxone, the stuff that brings you back if you go out
NARCAN(naloxone HCl) NASAL SPRAY 4 mg
Two Pack
Why don't you go first so I can make sure you're good, then you can watch out for me. If things go wrong you just spray it up my nose.
Cool. Thanks man.

YOU DESERVE QUALITY HEALTHCARE!

TIPS FOR HOW TO TALK TO YOUR DOCTOR TO GET THE MOST OUT OF YOUR APPOINTMENT

It can feel stressful to go to the doctor. You may be concerned that your doctor will judge you for using drugs or that you won't get quality healthcare. We are working on improving this at VA.

HERE ARE THINGS YOU CAN DO:

1. Prepare for your visits

- Think ahead about your goals for the appointment. What is important for you to address?
- Make a list of questions you have and identify the ones that are a top priority.
- Write down concerns you have about symptoms or changes in your health. You may be asked when these started and how much they affect your daily life.
- Take care of any drug-related needs before the appointment. You want to be alert and not in withdrawal. You also don't want to be too high.
- Bring your IDs and/or medical insurance card if you have one.

2. What you share is up to you

- Talking about your drug use is a personal choice.
- It may take time to build a trusting relationship with your doctor before you feel comfortable talking openly- and that's okay!
- Answering questions about your drug use may help your doctor understand some of your health concerns. This can help get you connected to the right healthcare or treatments.
- If your doctor seems too focused on your drug use, it is OK to respectfully remind them that this is not your issue of concern.
- If you're interested in starting treatment to cut down on your drug use, your doctor can help. But you don't have to want to stop using to get quality healthcare

3. Understand your next steps

- Ask any questions about your doctor's recommendations, such as medications, exercises, tests, imaging, or blood work.
- Ask for assistance with making follow up appointments.
- Ask for next steps and treatment recommendations in writing.

4. People who can help

- Every VA Medical Center has a Patient Advocate. Reach out and talk with the Advocate if you have questions or concerns about your care. They can help.

Resources to Help Keep you Safe While Using

Syringes

Most states have Syringe Service Programs (SSPs) where you can access new supplies, including sterile needles, syringes, cookers, cottons, and naloxone.
Scan the code to find the closest SSP or ask your doctor if your VA provides supplies

Overdose Prevention

Always carry naloxone (Narcan ®). You can ask your VA provider for a prescription or find free naloxone in your community.

Never use alone. If you are not able to use with other people around, call the Never Use Alone Hotline at 1-800-484-3731—Program it into your phone!

Call 911 if you are with someone who overdoses.
There are laws that protect you from criminal penalties.

Reduce Use

There are many resources available to help you reduce your use of drugs, if you're interested. If you are using opioids, medications like Buprenorphine and Methadone can help. Scan the code to learn more.

WARNING SIGNS THAT YOU SHOULD SEE A DOCTOR

- Fever, Chills, Sweats
- Fatigue
- Shortness of Breath
- Severe Pain in Your Back
- Severe Muscle or Joint Pain
- Difficulty Moving Your Muscles
- Abscess
- Rash or Red Spots on Hands or Feet
- Slurred Speech, Weakness, Facial Droop

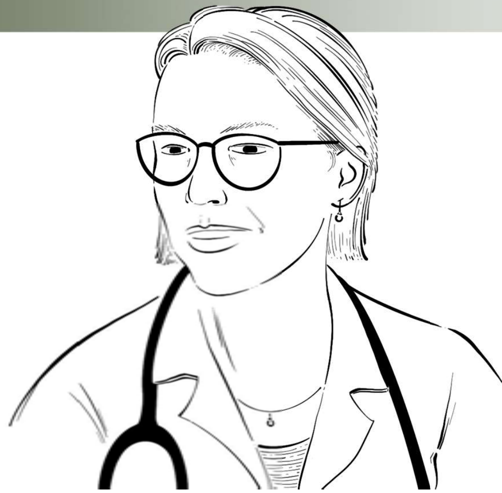

VA / U.S. Department of Veterans Affairs

This booklet was produced with support from:
VA Health Services Research and Development
Innovation Initiative Award # I01 HX003191 and SWIFT Grant

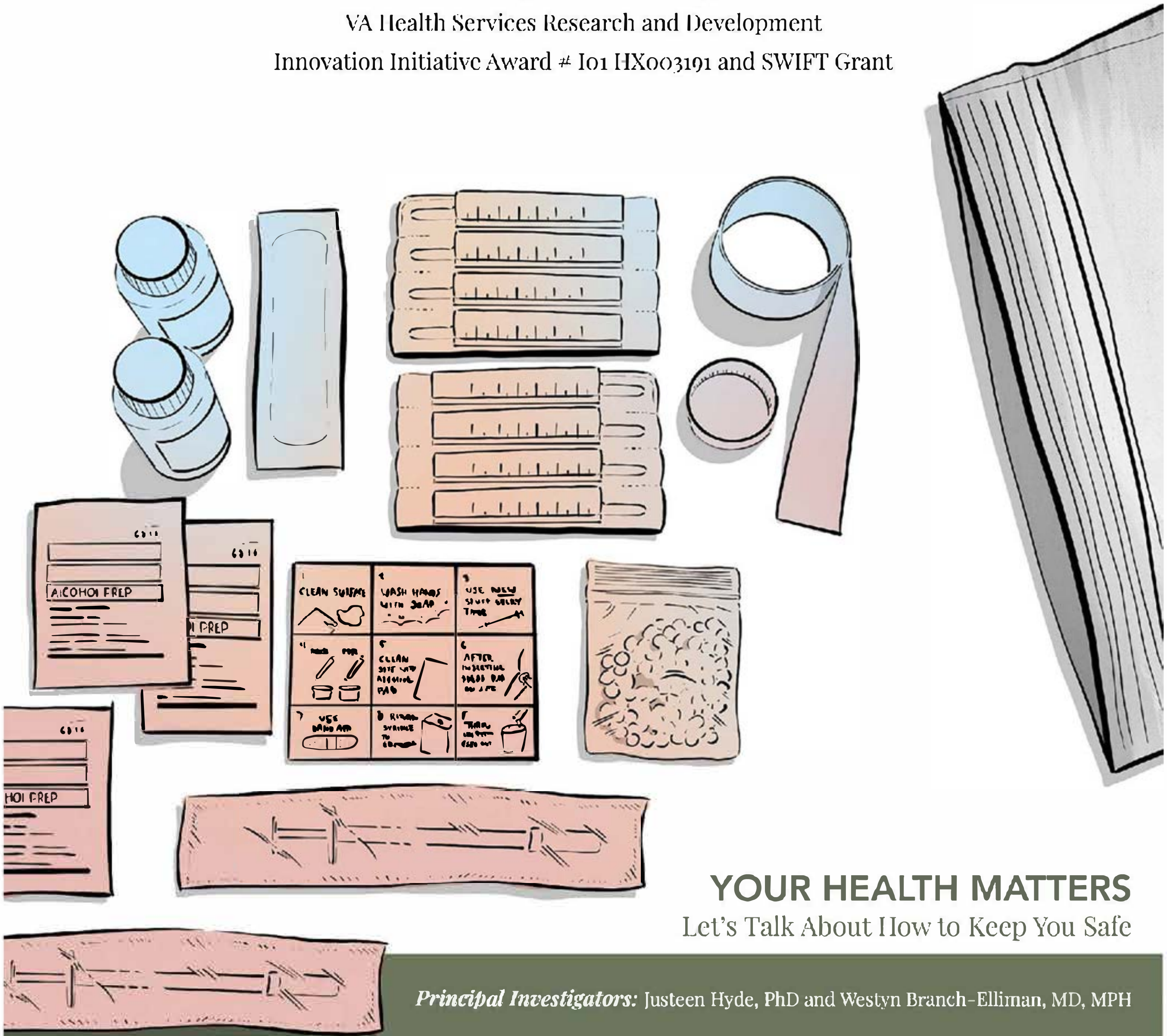

YOUR HEALTH MATTERS

Let's Talk About How to Keep You Safe

Principal Investigators: Justeen Hyde, PhD and Westyn Branch-Elliman, MD, MPH

Co-Designed by a team of Veterans, Clinicians, Community Harm Reduction Specialists, and Researchers:
Jacqueline Boudreau, Kenneth Boyd, Westyn Branch-Elliman, Rodney Calloway, Wendell Calloway, Juan Cortez, Elizabeth Dinges, Allen Gifford, Leah Harvey, Gary Hayes, Minh Ho, Justeen Hyde, Julian Saunders, Agustin Serrano, Samantha Sliwinski

Illustrated by: Tyler Thompson
